# Machine learning-based viewers’ preference prediction on social awareness advertisements using EEG

**DOI:** 10.3389/fnhum.2025.1542574

**Published:** 2025-06-13

**Authors:** Farhan Ishtiaque, Mohammad Tohidul Islam Miya, Fazla Rabbi Mashrur, Khandoker Mahmudur Rahman, Ravi Vaidyanathan, Syed Ferhat Anwar, Farhana Sarker, Noor Azman Ali, Huam Hon Tat, Khondaker A. Mamun

**Affiliations:** ^1^AIMS Lab, IIRIC, UIU, Dhaka, Bangladesh; ^2^School of Business and Economics, United International University, Dhaka, Bangladesh; ^3^Department of Biomedical Engineering, University of Rochester, Rochester, NY, United States; ^4^Department of Mechanical Engineering, Imperial College London, London, United Kingdom; ^5^Office of the Vice Chancellor, BRAC University, Dhaka, Bangladesh; ^6^Center for Computational & Data Sciences (CCDS) and Department of Computer Science and Engineering, Independent University, Bangladesh, Dhaka, Bangladesh; ^7^Putra Business School (PBS), Universiti Putra Malaysia (UPM), Selangor, Malaysia; ^8^Faculty of Business, City University of Macau, Taipa, Macau, China; ^9^Department of CSE, United International University, Dhaka, Bangladesh

**Keywords:** neuromarketing, EEG, consumer preference prediction, machine learning, consumer neuroscience

## Abstract

**Introduction:**

One of the most promising applications of neuromarketing is to predict true consumer preference for advertisements to quantify their efficacy. Researchers have already established such neuromarketing systems for static advertisements and e-commerce products. However, more research is required to develop such a system for dynamic advertisements. In this study, we predicted consumer preference for awareness advertisements and explored neural clues that may generate new insights on how we can evaluate advertisements using neuromarketing techniques.

**Methods:**

We took 4 awareness topics and selected 2 advertisements from each topic, using 2 types of storytelling (‘shock’ and ‘comic’), giving us a total of 8 advertisements. We prepared a custom 14-channel EEG dataset of 20 individuals watching these ads, along with their preferences and other self-reported measures. Machine learning was used to perform binary classification on viewers’ preferences. Additionally, other markers, such as engagement index and alpha activity, were studied.

**Result:**

The highest average accuracy of 72% was achieved using the leave-one-ad-out method. Further analysis shows that the engagement index (beta/alpha + theta) or (beta/alpha) is an important indicator of self-reported ratings for these advertisements, which have been reported previously.

**Discussion:**

Our ML model outperforms the current state of the art in terms of model accuracy. Additionally, awareness advertisements were used for the first time for such a task since these advertisements are free from any sort of product or brand bias. This ensures that the preferences of the advertisements were solely on the design and storytelling.

## 1 Introduction

Neuromarketing is a multidisciplinary field that combines principles from neuroscience, psychology, and marketing to gain insights into consumer behavior and preferences (Lee et al., 2007). It involves using various neuroscientific techniques to understand how the human brain responds to marketing stimuli, such as advertisements, packaging, products, etc. Companies are spending more than $1 trillion every year on advertisement and marketing (Worldwide Advertising, 2025). In this competitive space, it is very important to understand what consumers like, their behavior, and trends. Marketing and advertising research are the only way to achieve these. However, traditional research methods fall short of figuring out consumers’ true intentions using questionnaires, surveys, and focus group discussions (Hulland et al., 2018) as these methods are incapable of finding consumers’ true intentions (Rawnaque et al., 2020). On the other hand, Neuromarketing offers a more comprehensive analysis of consumer responses using various tools.

In the last two decades, researchers used many approaches for studying consumer decision-making and preference. Facial expression (Filipović et al., 2020), functional magnetic resonance imaging fMRI (Hsu and Cheng, 2018), electroencephalography (EEG; Golnar-Nik et al., 2019), magnetoencephalography (MEG; Hege et al., 2014), eye-tracking (Khushaba et al., 2017) etc. have been used as neuromarketing methods. Among these methods, fMRI, EEG, and MEG are capable of looking at the direct brain response and generating valuable insights. At first, researchers were primarily interested in using fMRI due to its high spatial resolution. However, the technology is inaccessible for practical use of neuromarketing. That’s why researchers are widely using EEG nowadays. EEG is defined as the brain’s electrical activity recorded using electrodes. EEG can be obtained invasively or non-invasively. Noninvasive EEG is the most common type of EEG which is used in neuromarketing. Among all the techniques that measure the brain’s activity, EEG provides excellent temporal resolution with decent spatial resolution, portability, and practicality.

Currently, several neuromarketing techniques are being practiced across the world with great success for advertisement testing and consumer research. It has been used to understand consumer behavior (Halkiopoulos et al., 2022), estimate comfortable prices (Herbes et al., 2015), advertisement testing (Mashrur et al., 2021, 2022; Telpaz et al., 2015), user interface design (González-Mena et al., 2022), etc. The possibilities are limitless as new solutions are popping up now and then as EEG, Eye-tracking, and othertechnologies are becoming cheaper and more accessible (Adeola et al., 2022).

Among many use cases, one of the most valuable uses of neuromarketing is to analyze and predict consumer preferences using EEG. In the last decade, several studies have achieved success in predicting consumer preference for static advertisements using EEG. Khushaba et al. (2017) analyzed the change of spectral activity of EEG while participants were asked to select the preferred version of crackers. 57 variants of crackers of different toppings, shapes, and sizes were used. Like/dislike classification on shoe images was studied by Yilmaz et al. (2014) to predict consumer preference from EEG signals. A similar study was performed by Yadava et al. (2017). They used 42 photographs of different items to classify preferences using the Hidden Markov Model. In our previous study (Mashrur et al., 2022), we used images of products, promotions, and endorsements of different items to classify preference as affective attitude and purchase intention. Similar to these most studies have focused on static advertisements for preference prediction and have established efficient methods to do that. However, preference prediction for video advertisements is still behind. In this method, participants wear EEG caps equipped with electrodes that measure electrical brain activity. When exposed to video advertisements, their brain responses are recorded, providing valuable data on the emotional and cognitive impact of the ad. By studying EEG data, researchers can identify moments in the advertisement where viewers experience heightened attention, emotional engagement, or cognitive processing (Ohme et al., 2009). This information helps advertisers tailor their content to be more engaging and persuasive, ultimately leading to more effective advertising campaigns (Robaina-Calderín and Martín-Santana, 2021). Neuromarketing, and specifically EEG analysis of advertisements, offers a deeper understanding of how consumers respond to marketing content, enabling businesses to create more compelling and impactful advertising strategies (Suomala, 2018). Video advertisements play a crucial role in modern marketing strategies, offering a dynamic and engaging medium to communicate with audiences (Munsch, 2021). They have the power to evoke emotions, tell stories, and convey brand messages effectively, ultimately driving brand awareness, engagement, and conversion. Emotionally resonant video advertisements are more likely to be remembered by consumers (Wiese et al., 2020). Several studies have delved into the connection between neuromarketing insights from video advertisements and subsequent consumer decision-making. Yang et al. (2015) compared the EEG of participants watching TV commercials with the EEG of participants watching ground truth videos of happiness, anger, and surprise to figure out important features for detecting these behavioral traits. Cherubino et al. (2019) analyzed the withdrawal index and engagement index to figure out the real-time consumer response during TV commercial perusal. These studies have developed the idea of linking EEG measures to consumer preference. This led to many researches, that tried to predict consumer preference for video advertisements using EEG.

Initial studies on consumer preference prediction based on EEG were performed for music videos. Moon et al. (2013) and Hadjidimitriou and Hadjileontiadis (2012) performed classification on EEG data of participants watching music videos and predicted preference. Moon et al. (2013) used the KNN and SVM approach on 84 features extracted from 21-channel EEG to predict consumer preference and achieved an accuracy of 97.39%. Hadjidimitriou and Hadjileontiadis (2012) performed a similar classification using KNN and achieved an accuracy of 86.52%. In terms of classical advertising, Soria Morillo et al. (2016) used 14 TV commercials to predict preference and achieved 75% accuracy using a tree-based machine learning algorithm. Hakim et al. (2018) used 6 food advertisements to predict preference and achieved 68.5% accuracy. In all of these studies, traditional ads were used for classification (Table 1).

**Table 1:** Summary of studies conducted on preference prediction on video.

Study	Video type	Classification model	Number of features	Class	Best classification accuracy
Moon et al. (2013)	Music Video	KNN, SVM	84 (21-channel EEG)	1. Most preferred 2. Preferred 3. Less preferred 4. Least preferred	97.39%
Hadjidimitriou and Hadjileontiadis (2012)	Music Video	KNN	270 (14-channel EEG)	1. Like 2. Dislike	86.52%
Soria Morillo et al. (2016)	14 TV commercials	ANN, DT	1,064 (14-channel EEG)	1. Like 2. Dislike	75%
Hakim et al. (2018)	6 food commercials	SVM, KNN, Tree, LR	25 (8- channel EEG)	1. Like 2. Dislike	68.5%
Kosonogov et al. (2023)	8 short films	SVM, KNN, LR	519 (18-channel)	1. HR 2. LR	70%

Traditional advertisements often present challenges for neuromarketing research due to the presence of inherent biases associated with brands, celebrities, or specific products (Dey and Gayathri, 2021; Simmonds et al., 2020). When individuals are exposed to well-known brands or recognizable celebrities in advertisements, their pre-existing attitudes and perceptions can significantly influence their neural responses. This bias can obscure the genuine neurological reactions to the advertising content itself, making it difficult to isolate the impact of the advertisement’s design, narrative, or emotional appeal (Alsharif and Isa, 2025). In contrast, neuromarketing research seeks to uncover the intrinsic, subconscious reactions that individuals have to marketing stimuli. To mitigate these biases, researchers often turn to more neutral or controlled stimuli, using experimental content that lacks prior associations to allow for a clearer understanding of how different aspects of advertising impact consumer decision-making and behavior. By doing so, neuromarketing can offer a more accurate and insightful perspective on the underlying cognitive and emotional responses that drive consumer choices and preferences.

In order to achieve a neutral environment, researchers have used movie trailers, short films, or documentaries as forms of stimuli to uncover important information about the brain introducing a new area of Neurocinematics research. A research by Boksem and Smidts (2015) showed how the beta activity of the brain can be an effective indicator of likability and predict the efficacy of movie trailers. Similar work done by Kosonogov et al. (2023) on short films has introduced the alpha and beta activity to be an effective attribute of engagement which can help predict the efficacy of such media.

Awareness advertisements, often referred to as public service announcements (PSAs) or social awareness campaigns, are a powerful and influential form of media content designed to inform, educate, and create awareness about important social, environmental, or health-related issues (O’Keefe and Reid, 1990). These advertisements are typically non-commercial in nature and focus on raising public consciousness rather than promoting a specific product or service. Awareness advertisements tackle a wide range of subjects, such as public health initiatives, environmental conservation, social justice causes, and community safety. They employ various media platforms, including television, radio, print, digital media, and social networks, to reach a broad audience. The primary goal of awareness advertisements is to convey a meaningful message and inspire positive action among the general population, addressing critical societal concerns and promoting positive change. These campaigns often collaborate with nonprofit organizations, government agencies, and advocacy groups to maximize their impact and foster a sense of shared responsibility for the well-being of society and the world at large. The effectiveness of such advertisements is very important for socio-economic stability. The effectiveness of awareness advertisement using EEG, has not yet been rigorously studied in the neuromarketing or neurocinematics field. Cartocci et al. (2017) explored the efficacy of such PSA using EEG, GSR, and Heart rate. However, the study was limited to finding key aspects of the data to best explain the outcome variable. Martinez-Levy et al. (2022) studied the effects of text placements and small design changes in PSA ads using EEG and other matrices, however, a more generalized study for consumer preference is still required. So, in this study, we aim to predict the preference for awareness advertisements using machine learning analyze the effectiveness of awareness ads, and figure out valuable neural information while people experience these awareness ads.

In this study, we collected 14 channel EEG data from 20 subjects while they were watching 8 different awareness advertisements. After data collection, we processed the data to remove artifacts. Then we extracted several EEG-based features and performed multiple data analysis techniques to depict valuable information that will help us to evaluate the advertisement. Finally, we applied machine learning to predict the binary rating of the advertisements. Here are the major contributions of the study,

- To the author’s best knowledge, this is the first study that uses awareness advertisement and machine learning for consumer preference prediction and finding key neural markers.
- In this study, we found the engagement index (beta/alpha + theta) to be a potential candidate for a neural marker that has a significant correlation with ad rating.
- We found the alpha activity to be an indicator of discomfort while watching some ads and can be used as a discomfort index.
- We proved that machine learning models can be used to predict the rating of such advertisements based on EEG features.

## 2 Materials

### 2.1 Participants

Twenty healthy young volunteers (age: 24 ± 5) participated in this study with no history of neurological or mental disorders. Before the study, according to the Helsinki Declaration and Neuromarketing Science and Business Association Code of Ethics (NMSBA), all participants provided their consent. The study is also approved by the United International University, Institutional Research Ethics Board committee. After the data collection process data anonymization was performed, removing any personal information from the data. Additionally, the data is stored locally using standardized methods mitigating any potential data breach.

### 2.2 EEG device

**Figure 1:**
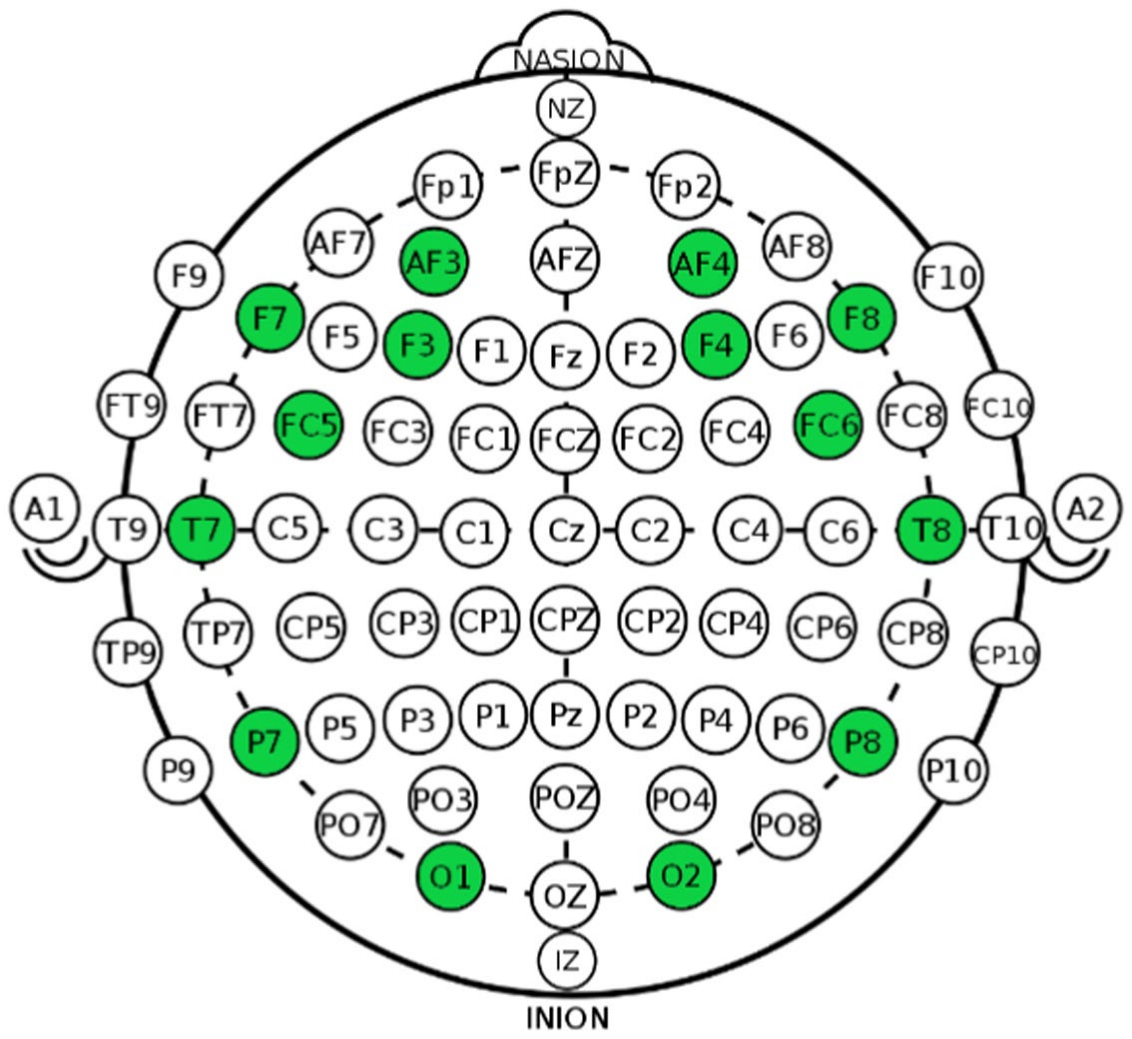
Channel locations of emotiv epoch. It collects data from pre-frontal (2), frontal (6), temporal (2), parietal (2) and occipital (2) regions of the brain.

In this study, we used the Emotiv Epoc X from Emotiv for data collection. Emotiv Epoc X is a 14-channel, semi-dry electrode EEG device. This device is used in many neuromarketing studies including (Alsharif and Isa, 2025; Mashrur et al., 2022; Pal et al., 2021). It is a widely used EEG data collection device in research as it covers a wide region of the brain using 14 channels. It is also relatively easy to set up which is perfect for neuromarketing applications. The channel location of the device is shown in Figure 1.

### 2.3 Stimuli description

**Figure 2:**
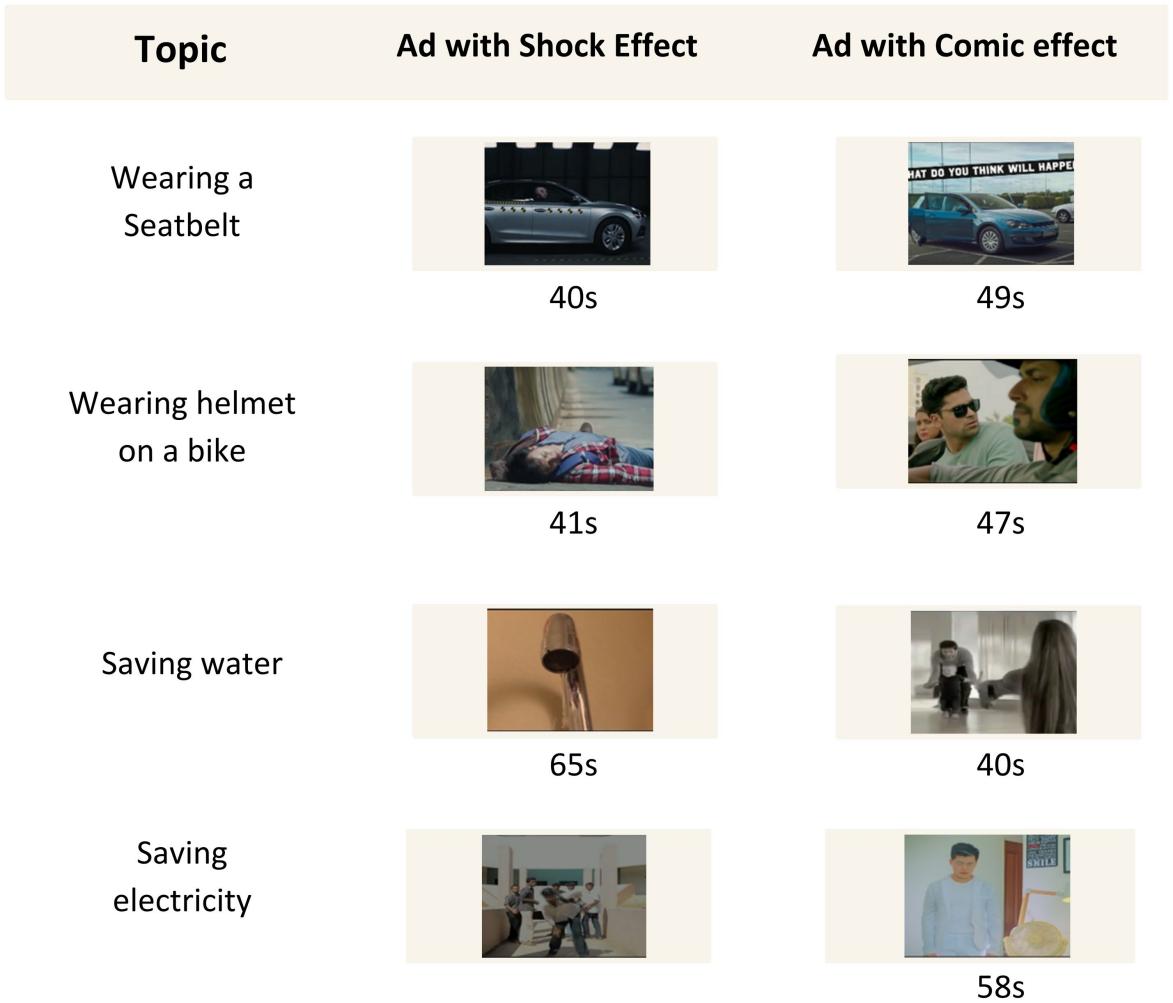
List of stimuli used in this study along with duration. We used eight ads from four categories. Two types of ads for each category were selected.

A total of 8 (Duration: Average 49 ± 9 s) awareness videos of 4 different areas were taken for this study. The areas are, “Wearing Seatbelt on a Car,” “Wearing Helmet on a Bike,” “Saving Water” and “Saving Electricity.” 2 awareness videos of each area also have two types of effects, e.g., “Comic Effect” and “Shock Effect” (endorsed by two marketing experts). Figure 2 shows the stimuli list.

### 2.4 Participants’ response description

A wide range of responses were taken from the participants during the experiment. Before the presentation of stimuli, the awareness level of the participants for each topic was recorded. During the presentation of stimuli, the participants were asked to rate each ad based on their likeness. After the presentation, another survey was taken where the participants’ awareness level was taken again along with the ad shareability. Table 2 summarizes the responses taken from the participants.

**Table 2:** Summary of responses taken from the participants during the experiment.

Survey form	Information collected
Pre-stimuli survey	Name, Age, Gender, Handedness Product Relatability Current Awareness about seatbelts, helmets, and water and electricity saving (On a scale of 10)
Mid stimuli response	Rating of advertisement (Out of 10) on “Likeness of how well the message was conveyed”
Post stimuli survey	Current Awareness about seatbelts, helmets, and water and electricity saving (On a scale of 10) Ad shareability (On a scale of 10)

The pre-stimuli survey was done before the participants were shown stimuli and wore the EEG device. Then during the stimuli presentation, the mid stimuli responses were taken. Lastly, after the stimuli presentation, the participants filled out the post-stimuli survey.

### 2.5 Data collection

**Figure 3:**
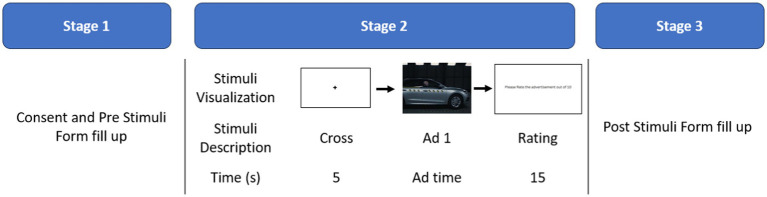
Three stages of data collection. Participants filled up the pre-stimulus form and consent form in stage 1. In stage 2, the videos were shown sequentially and a rating was taken. Stage 2 was repeated 8 times for 8 advertisements. Lastly, in stage 3 the post stimuli form was given.

The data collection procedure can be divided into three stages; described in Figure 3. In the first stage, the participants were seated in front of a screen. The participants were asked to sign a written consent form and then fill out the “Pre-Stimuli Survey” form. They were also given a small instruction about the experiment in this stage.

In the second stage, an attendant helped the participants to wear the EEG device and then the stimuli were shown in a random order. A black cross in front of a white screen was shown for 5 s before each stimulus to help the participants focus. After each stimulus, the participants were asked to rate the advertisement (On a scale of 1 to 10) about how well it conveyed the message on a sheet of paper using a pen. The stimuli were shown using PsychoPy (Peirce, 2007). To align EEG data with onset and duration of the stimuli we used the Emotiv plugin for PsychoPy. This plugin adds an extra field (14 channel + 1 marker field) in the collected data which contains 0 for each row and adds 1 whenever the marker was triggered. In PsychoPy, the markers were triggered at the start and end of each stimulus.

Finally, in the third stage, the participants were asked to fill out the “Post Stimuli Survey” form.

## 3 Methods

### 3.1 Data pre-processing

For pre-processing the collected EEG signal, MATLAB and EEGLAB are utilized. A zero-phased, 3rd order bandpass-Butterworth filter with a frequency range of 0.5–70 Hz is first applied to the signals, with the goal of removing noise at both high and low-frequency ranges. After that, a notch filter at 50 Hz is applied in order to get rid of the noise from the powerline. ICA is used to remove ocular artifacts from the data, following, the automated subspace reconstruction (ASR) tool of EEGLAB was used to remove other artifacts caused by movements. For ICA we used the ‘runica’ algorithm of EEGLAB which is built on the Information-Maximization Approach introduced by Bell and Sejnowski (1995). The independent components that contained ocular artifact were identified by visual inspection for each data. In the final step, the data are normalized by first subtracting the average of all data points from each point, and then dividing the resulting value by the standard deviation. The data was organized in a structured time series vector X(t).

### 3.2 Feature extraction

A total of 470 multi-domain features were extracted from each channel of the processed EEG signal. These features can be categorized as time domain, frequency domain, and time-frequency domain features.

The full feature list that is used in this work is provided in Supplementary material 1. Time domain features, also known as statistical features were introduced by Golnar-Nik et al. (2019) and Yadava et al. (2017) in neuromarketing works. We expanded these statistical features further. Therefore, standard statistical features were included (f3-f30 in Supp 1). Some previous studies in consumer neuroscience conclude dispersion is an important feature, which is why f12-f20 were included in the study (Ahammad et al., 2014; Inuso et al., 2007; Islam et al., 2013; Angelika and Buss Martin, 2014). In addition, previous research suggests that frequency band oscillation and spectral changes are important to consider when analyzing EEG data for decision-making, attention, and consumer choice; as a result, we made use of a variety of spectral features, f31 to f41, in this investigation (Foxe and Snyder, 2011; Mashrur et al., 2021; Anders et al., 2013; Telpaz et al., 2015).

**Figure 4:**
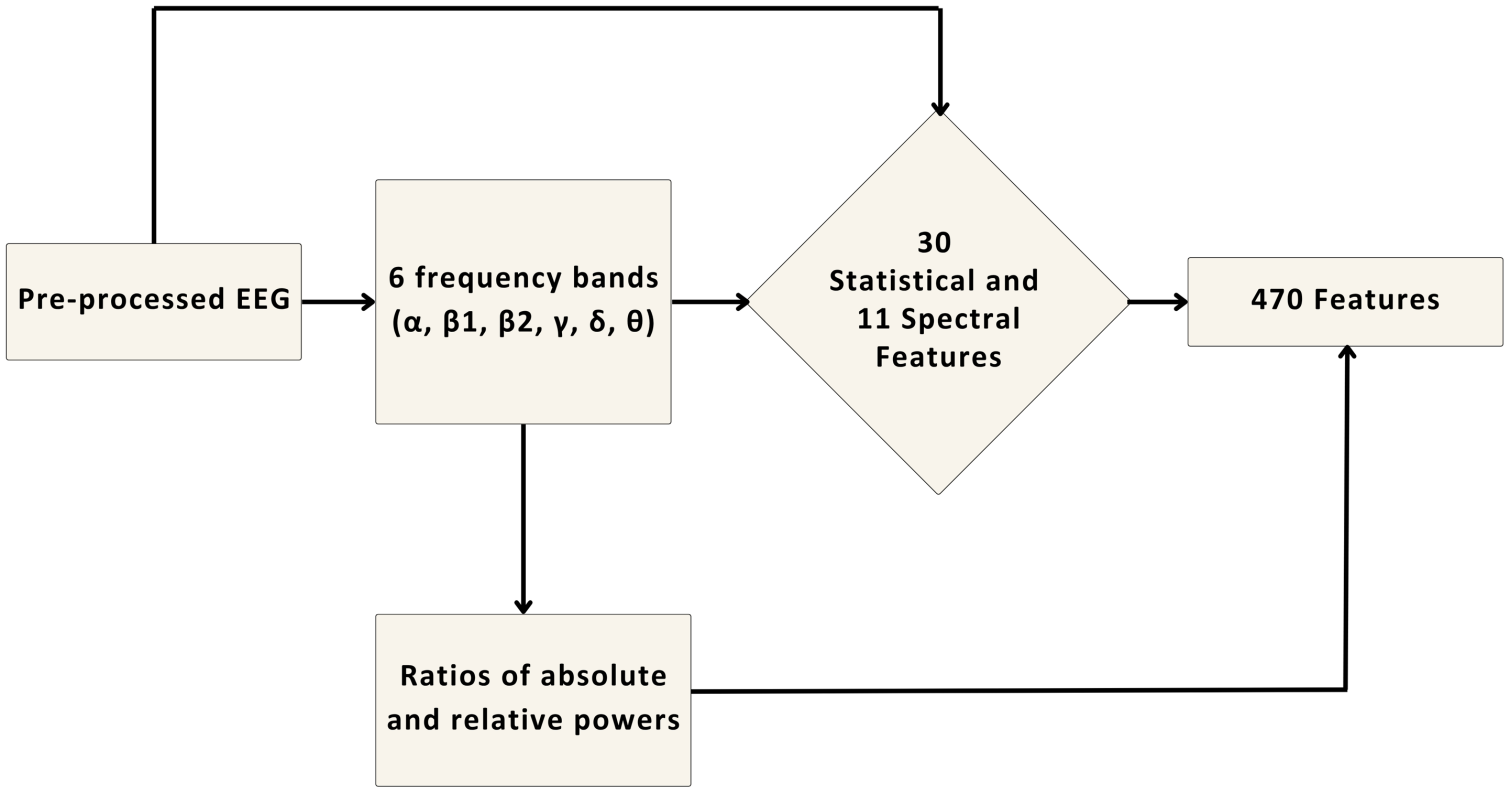
The pre-processed EEG was used to extract 41 features first. Then it was decomposed into 6 frequency bands and those features were extracted again from these six bands. Finally, the ratio of average and relative power of all bands was also taken to create 470 features from each channel.

For feature extraction, the first 30 statistical and 11 spectral features were calculated for X(t) which were denoted as time domain features and frequency domain features, respectively. Then X(t) was decomposed into six frequency bands namely, alpha (8–12 Hz), beta_1 (12–20 Hz), beta_2 (20–32 Hz), gamma (32–64 Hz), theta (4–8 Hz) and delta (0–4 Hz) and all those features have been extracted from all six frequency bands. Finally, the ratios of absolute and relative powers have been taken, completing all time-frequency domain features. The total number of features at the end is summed up to be 470 for each channel. The final feature set consisted of a total of 6,580 (14 × 470) features. A summary of the feature extraction process is given in Figure 4.

### 3.3 Data analysis

We correlated ad ratings and engagement index (beta/alpha) and (beta/(alpha + theta)) across all channels. Benjamini-Hochberg correction was applied for correlations of each individual channel (Thissen et al., 2002). This engagement index was previously defined in Pope et al. (1995) and Freeman et al. (1999) and was found to be a significant indicator of effective rating (Kosonogov et al., 2023). The baseline for this index in neuromarketing has been set by Boksem and Smidts (2015) and Cohen et al. (2007) as they found beta activity to be related to preference. Kosonogov et al. (2023) used this to predict film ratings.

**Figure 5:**
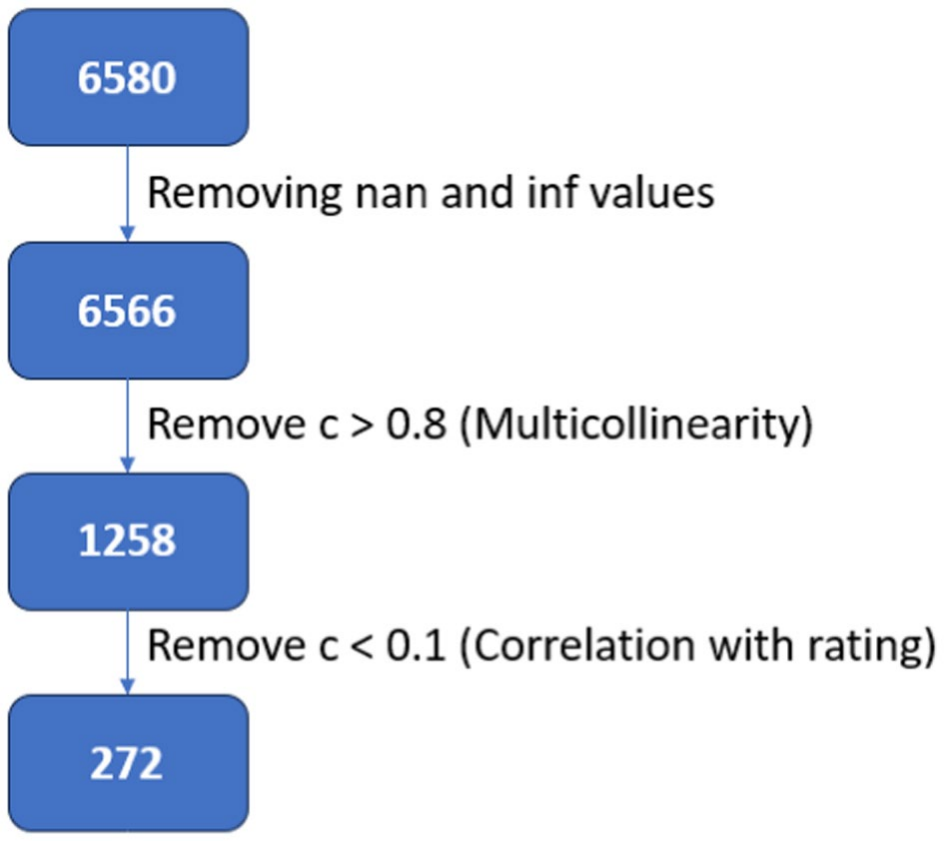
Feature selection process using correlation based filtering.

For binary classification of ad rating, based on EEG data, first we performed feature selection. The feature set was reduced from 6,580 to 272 using an ensemble method. First, the feature set was reduced from 6,580 to 1,258. During this process, all features were correlated with each other. If two features have a correlation coefficient greater than 0.8, one of these two is kept and the other one is removed. This method was introduced by Yu and Liu (2003) and is widely used for feature elimination (Khaleghi et al., 2015; Şen et al., 2014; Sereshkeh et al., 2017). After that, these 1,258 features were correlated with normalized ad ratings, and only those features whose correlation coefficients were found (>0.1) were kept. This way the feature set was reduced to 272 (Figure 5). Then the 272 features were further reduced using SVM-based recursive feature elimination. This was performed during the classification, within the cross-validation loops for increasing robustness.

For binary classification, we normalized the ad rating across participants and split the dataset into two classes, high rating (r > = 0.5) and low rating (*r* < 0.5). The sample size of high rating was 108 and low rating was 52. After that, a Support Vector Machine with optimized parameters (C = 10, g = 0.01, and kernel = “rbf”) was used to classify the dataset using leave-one-ad-out (LOAO) method. For this method, the classification was done 8 times, for each classification 1 ad was used for testing, and the other 7 was used for training. During classification hyperparameters of the SVM model was tuned using grid search method. Random Forest, Decision Tree, and Logistic Regression were also used to classify the data for comparison. For each LOAO iteration, feature selection on 272 features was performed using SVM-RFE. We used SVM with linear kernel and optimized hyperparameters (kernel = ‘Linear’, C = 0.25). 4-fold cross validation was performed during each LOAO iteration. The hyperparameters for this step was optimized using a nested grid search method. Initial grid was, [Number of CV loops: 3, 4, 5] and [C: 0.01, 0.1, 1, 10, 100] and then on the 2^nd^ iteration, [C: 0.1, 0.15, 0.2, 0.25, 0.3, 0.35] for each CV loops.

### 3.4 Evaluation metrics

We used three parameters to evaluate our machine learning models. These are,

Accuracy (acc): Accuracy is the overall performance of the model. It is the percentage of correctly predicted ratings among all samples of test data. Accuracy is calculated using the following formula,


(1) $$\text{Accuracy}=\frac{\mathrm{Tp}+\mathrm{Tn}}{\mathrm{Tp}+\mathrm{Fp}+\mathrm{Tn}+\mathrm{Fn}}$$

F1 Score (f1): F1 score is the model’s predictive power. F1 score is calculated using the following formula,


(2) $$F1\;\text{Score}=\frac{\mathrm{Tp}}{\mathrm{Tp}+(0.5)(\mathrm{Fp}+\mathrm{Fn})}$$

Area Under Curve (auc): AUC summarizes the model’s ability to distinguish between positive and negative classes. It is calculated using the following formula,


(3) $$\mathrm{AUC}=\int_{0}^{1}(\mathrm{TPR})\;\mathrm{FPR}\;\text{dFPR}$$

In Equations 1–3, Tp = True Positive, Tn = True Negative, Fp = False Positive, Fn = False Negative, TPR = True Positive Rate (Recall), FPR = False Positive Rate.

## 4 Result and discussion

We tested several machine learning models for binary prediction of ad rating, based on the EEG data. We classified each film as low-rated (LR) or high-rated (HR). The binary classification result of the LOAO method is shown in Table 3.

**Table 3:** Accuracy of 4 classification models of the leave-one-ad-out method.

Ad No.	SVM_acc	SVM_f1	SVM_auc	RF_acc	RF_f1	RF_auc	DT_acc	DT_f1	DT_auc	LR_acc	LR_f1	LR_auc
1	0.55	0.67	0.59	0.45	0.62	0.50	0.45	0.56	0.48	0.50	0.64	0.55
2	0.90	0.95	NA	1.00	1.00	NA	0.55	0.71	NA	0.85	0.92	NA
3	0.35	0.48	0.47	0.35	0.52	0.50	0.45	0.48	0.51	0.30	0.46	0.43
4	0.90	0.94	0.84	0.80	0.89	0.50	0.70	0.80	0.63	0.85	0.90	0.81
5	0.45	0.59	0.45	0.50	0.67	0.50	0.35	0.43	0.35	0.45	0.56	0.45
6	0.90	0.92	0.88	0.60	0.75	0.50	0.70	0.77	0.67	0.85	0.88	0.83
7	0.90	0.94	0.72	0.90	0.95	0.50	0.65	0.77	0.58	0.90	0.94	0.72
8	0.80	0.89	0.50	0.65	0.77	0.50	0.45	0.59	0.38	0.60	0.71	0.56
Average	0.72	0.80	0.64	0.66	0.77	0.50	0.54	0.64	0.51	0.66	0.75	0.62

Testing Accuracy (acc), f1 score (f1), and Area Under the Curve score (auc) have been presented in the table. The AUC score for ad 2 was not calculated as all responses were from one particular class (HR).

The highest average prediction accuracy was achieved by SVM which is 0.72. Previously, (Kosonogov et al., 2023) performed similar classification and achieved a maximum average accuracy of 0.60. F1 score of our proposed model is also comparatively higher at 0.8 than of Kosnogov’s 0.6. However, auc score of our model at 0.64 was similar to their 0.62. Although the machine learning models are similar the difference in EEG features must have caused the improvement. We used a lot more features to start with and used SVM-RFE for feature selection which helped improve the prediction accuracy.

One of the reasons for SVM outperforming other models is because the classification is being performed in a small dataset. SVM is known for performing well in small dataset whereas models like Random Forest requires large data. Additionally, since our problem is nonlinear in nature, using SVM’s nonlinear kernel gave it an advantage. However, for ad 2 the AUC score was not calculated as all the participants rated this ad as HR.

**Figure 6:**
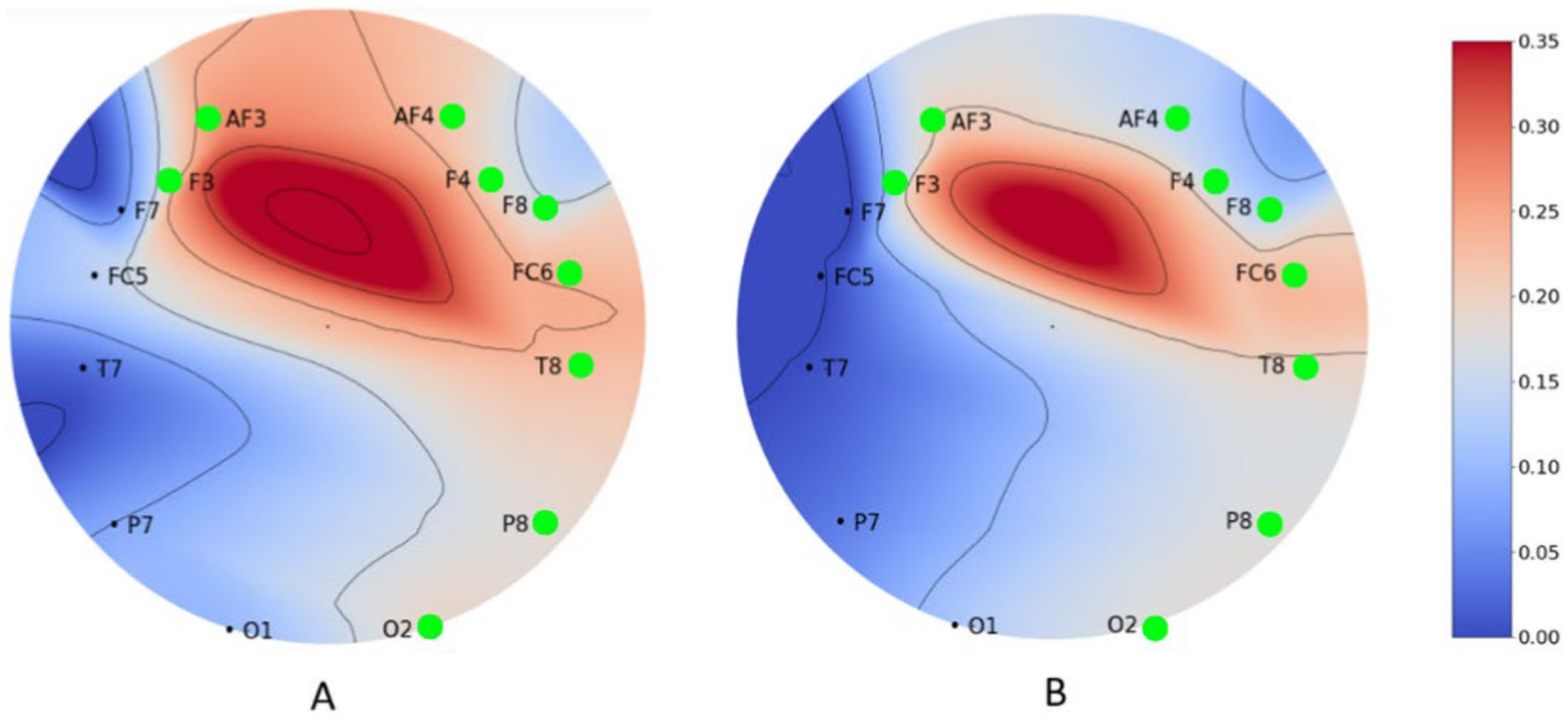
Correlation coefficient of engagement index. **(A)** (beta/alpha); **(B)** [beta/(alpha + theta)] of ad rating across all channels. The correlation is significant at the green marked locations.

Correlation analysis between EEG rhythms and ad ratings was performed. Both the engagement index, beta/(alpha+theta) and beta/alpha, correlated positively with ad ratings in 9 out of 14 channels (‘AF3’, ‘F3’, ‘O2’, ‘P8’, ‘T8’, ‘FC6’, ‘F4’, ‘F8’, ‘AF4’). The higher the index was, the larger the self-reported value was. Figure 6 shows the correlation coefficient of the engagement index, beta/alpha, and beta/(alpha+theta) across all channels. We conclude that the engagement index obtained from the frontal and central regions can be used as an indicator of ad ratings. The engagement index of the other regions does not significantly correlate with the ad rating hence proving them not worthwhile. Similarly, engagement index of central region was found significant in Kislov et al. (2022). The probable reason other brain regions are not giving significant correlation might be the due to the function of each region. Primarily, the frontal lobe is significant for emotional processing and decision making (Chayer and Freedman, 2001). Which is why the correlation might be stronger in this region. However, in Kosonogov et al. (2023) engagement index from the left parietal cortex was found to be significant which we did not find.

Alpha activity is reported as an important marker of stress or discomfort in many literatures (Katmah et al., 2021; Wen and Aris, 2020), evidence of which is also found by us in this experiment. It has been found that alpha activity increases when a person is comfortable and relaxed but decreases when a person is stressed or uncomfortable.

**Figure 7:**
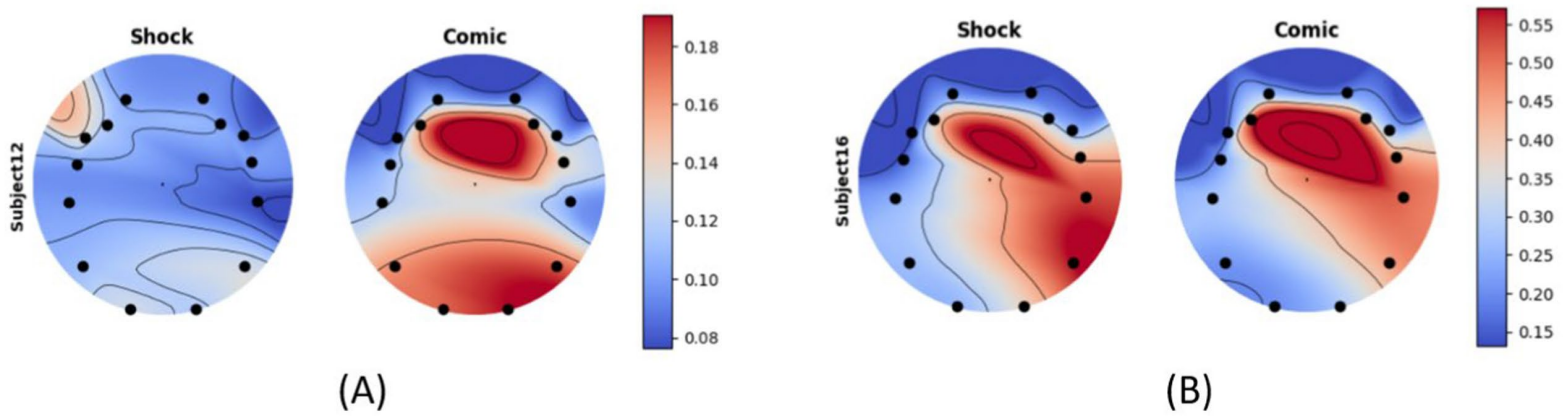
Alpha activity for shock vs. comic helmet ad of two different participants **(A)** subject 12 and **(B)** subject 16.

We report that the average alpha power across all channels is lower during the advertisement of the shock effect (mean 0.03747) than that of the comic effect (mean 0.04252). The difference between the alpha activities varies from person to person. Activity from two different subjects across all channels is shown in Figure 7.

**Figure 8:**
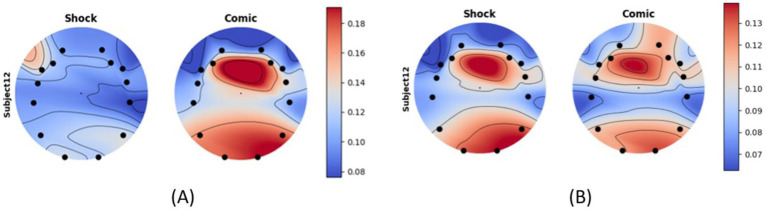
Alpha activity of subject 12 for **(A)** shock vs. comic of helmet ad and **(B)** shock vs. comic of water saving ad.

Furthermore, we found that the alpha activity difference of a subject depends on the type of advertisement. We found out that during the Shock effect ad of seatbelt and helmet, the person was in discomfort compared to the comic effect ad. Alpha activity across all channels of a single subject for helmet ad (shock) and water-saving ad (shock) is shown in Figure 8.

A comparison of alpha activity across all channels for all 20 participants is given in Supplementary material 2.

**Figure 9:**
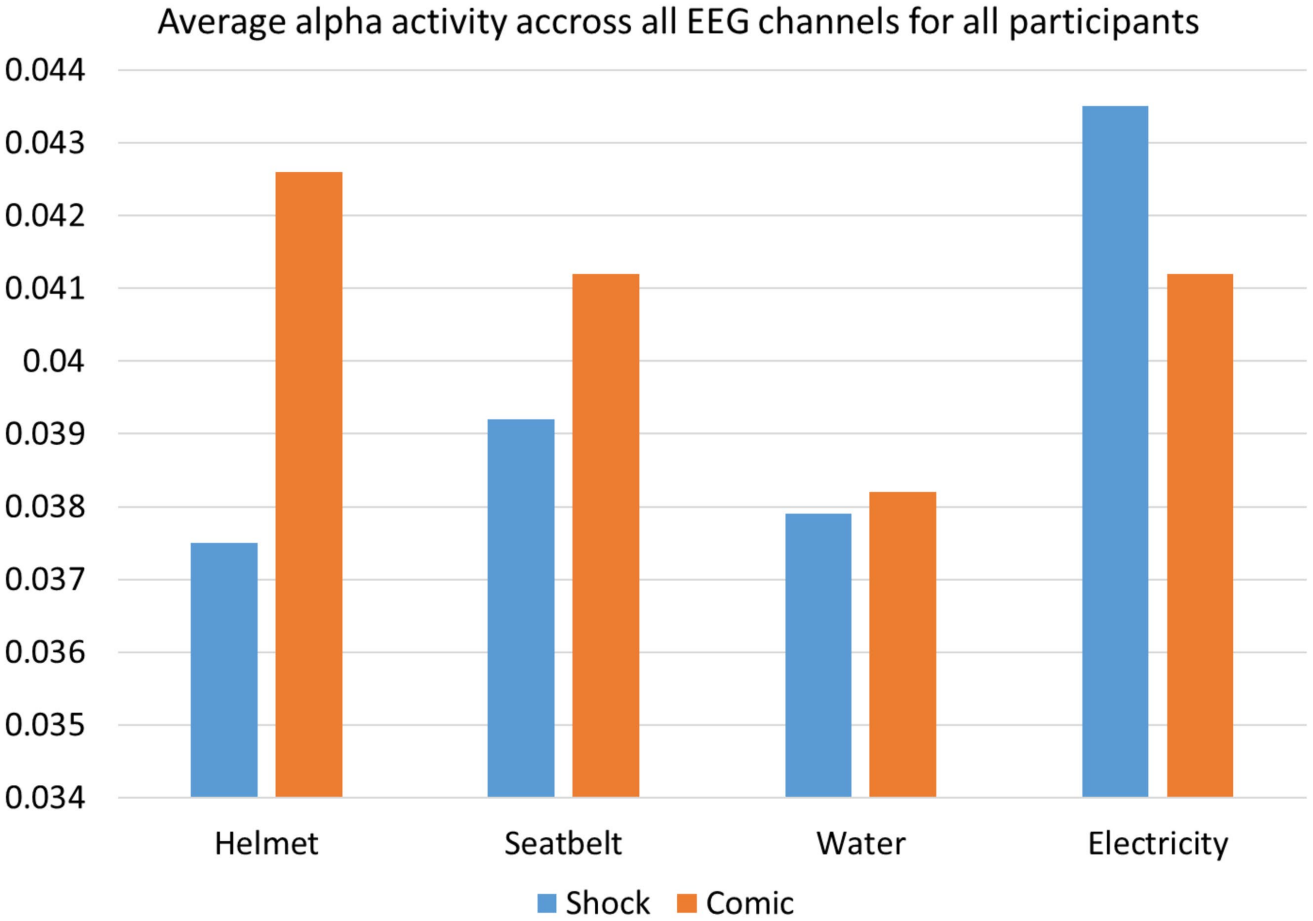
Average alpha activity of all participants for all advertisements shows that the difference between shock effect and comic effect is high for advertisements “Helmet” and “Seatbelt” and low for advertisements “Saving water” and “Saving electricity”.

The average alpha activity for all participants across all channels for all 8 ads has been shown in Figure 9. As discussed earlier we found that the difference between shock and comedic effect is prominent for seatbelt and helmet ads as the ads were graphic for both while for energy conservation the difference is not noteworthy. This marks alpha activity as an effective measure of stress and discomfort during advertisement-watching sessions which can be used for ad effectiveness analysis in the future.

## 5 Limitations and future work

One of the limitations of this study is having low variance in people’s self-reported ratings where more than 70% of responses were >5 and among them more than 90% were 10/10. The reason is that awareness ad by itself creates a positive impact on participants despite the fact that other aspects (story-telling, design, etc.) might lack efficacy. So, the participants’ response is skewed toward the high rating side. A more robust stimuli selection can increase the variance in the data which will further strengthen our claims. In the future, we like to perform similar studies with more variable contents.

During analysis we used the leave-one-ad-out approach for validation. Although it has its merits, it might not be as robust as the traditional leave-one-subject-out method. In future, we plan to collect a larger dataset and explore more methods.

Another limitation of this study is the lack of findings in terms of ad shareability metric. The ad shareability metric was also greatly skewed toward one response, more than 80% were >8 out of 10. Which limited our contribution in terms of ad shareability prediction and finding neural markers for it. In the future, we would like to improve our survey method to technically introduce more data variance in this regard.

Lastly, the sample size, 20 is on the lower end of similar studies. In subsequent studies, we would like to incorporate larger sample size with diverse demographic participants. Also, we used Emotiv EPOC X for data collection. Although, this device is very popular in neuromarketing studies, it is not as accurate as a medical grade EEG. However, we selected this device as it is currently the most suitable consumer-grade option for neuromarketing applications and is widely used.

## Data Availability

The raw data supporting the conclusions of this article will be made available by the authors, without undue reservation.
