# Supplementary material for: Machine learning-based viewers’ preference prediction on social awareness advertisements using EEG
Source: Front Hum Neurosci. 2025 Jun 13;19:1542574. doi: 10.3389/fnhum.2025.1542574 (PMC12202439; doi:10.3389/fnhum.2025.1542574)
Supplement: Supplementary file 1 [file Data_Sheet_1.PDF]

Index	Feature Name	Feature Description
f1	Average power	Average power of EEG using PSD and Welch's method
f2	Relative power	Ratio of Band power to total power
f3	Hjorth Mobility	Hjorth feature
f4	Hjorth Complexity	Hjorth Feature
f5	Skewness	Symmetricity of EEG signal
f6	Kurtosis	How much tailed the probability distribution of EEG signal
f7	Normalized First Difference	Self-similarities by one sample point
f8	Normalized Second Difference	Self-similarities by two sample points
f9	Arithmetic Mean	Mean of EEG signal
f10	Median Value	Median of EEG signal
f11	Maximum Value	Highest point of EEG signal
f12	Minimum Value	Lowest point of EEG signal
f13	Absolute Value of Square Root	Summation of square root of absolute EEG signal
f14	Modified Mean Absolute Value	Mean absolute value of EEG signal
f15	Interquartile Range	Difference between 75th percentiles & 25th percentiles
f16	Difference Variance Value	Difference between neighboring samples' variance value
f17	Difference Absolute Standard Deviation	Absolute value of difference between neighboring samples' standard deviation
f18	Mean Teager Energy	Mean of non-linear energy
f19	Log Energy Entropy	Non-normalized energy entropy
f20	Renyi Entropy	Non-linear entropy of signal
f21	Standard deviation	Standard deviation from mean
f22	Threshold Crossing	Threshold value crossing frequency
f23	Zero Crossing	Sign change frequency
f24	Coefficient of Variation	Dispersion Measurement
f25	Log Coefficient of Variation	Logarithmic dispersion Measurement
f26	Slope Sign Change	Slope sign change frequency
f27	Absolute Value of Cube	Sum of cube of absolute EEG
f28	Log Detector	Non-linear natural exponential measurement
f29	Cardinality	Number of distinct values
f30	Detrend fluctuation analysis	Non-linear parameter of auto-correlation properties
f31	Spectral Centroid	Barycenter of the spectrum
f32	Spectral Spread	Distribution of the spectrum around its average value
f33	Spectral Skewness	Measures of asymmetric distribution around signal's mean
f34	Spectral Kurtosis	Flatness distribution of spectrum around its mean value
f35	Spectral Entropy	Peakedness distribution of the spectrum
f36	Spectral Flatness	Noise like nature of the spectrum
f37	Spectral Crest	Sinusoidality of the spectrum
f38	Spectral Flux	Power spectrum change measurement
f39	Spectral Slope	Linear decreasing of the spectral amplitude
f40	Spectral Decrease	Decreasing of the spectral amplitude
f41	Spectral Rolloff Point	95th percentile of the spectral power distribution
