# Supplementary figures and images for: Machine learning-based viewers’ preference prediction on social awareness advertisements using EEG
Source: Front Hum Neurosci. 2025 Jun 13;19:1542574. doi: 10.3389/fnhum.2025.1542574 (PMC12202439; doi:10.3389/fnhum.2025.1542574)

## Helmet

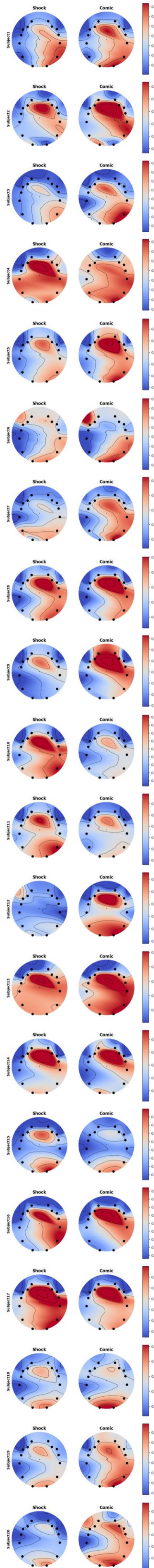

## Seatbelt

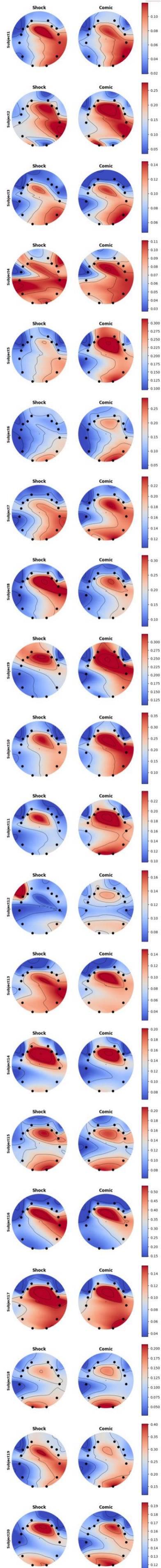

## Water

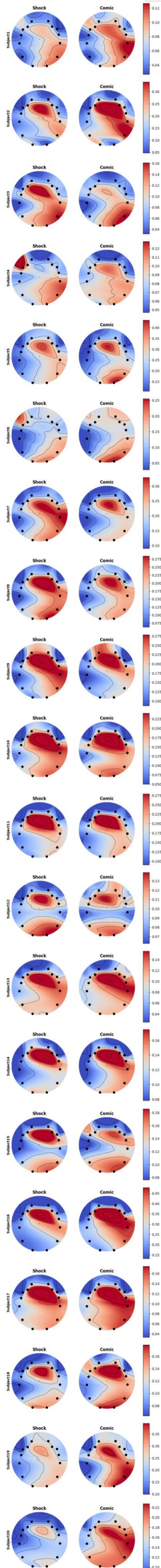

## Electricity

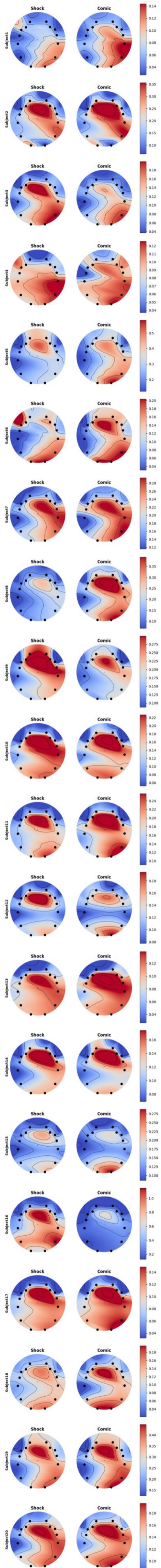

Supplement: Supplementary file 2 [file Data_Sheet_2.PDF]
